# Macular Telangiectasia Type 2 and Unilateral Subfoveal Vitelliform Lesion: A Longitudinal Follow-up By Multimodal Imaging

**DOI:** 10.22336/rjo.2026.24

**Published:** 2026

**Authors:** Hamit Ali, Omer Karti, Ziya Ayhan, Ayse Bozkurt Oflaz, Ali Osman Saatci

**Affiliations:** 1 Department of Ophthalmology, Dokuz Eylül University, İzmir, Türkiye; 2 Department of Ophthalmology, Selcuk University, Konya, Türkiye

**Keywords:** macular telangiectasia type 2, MacTel type 2, macula, macular atrophy, optical coherence tomography, vitelliform lesion, MacTel type 2 = Macular telangiectasia type 2, VL = Vitelliform lesion, OCT = Optical coherence tomography, RPE = retinal pigment epithelium

## Abstract

Macular telangiectasia type 2 (MacTel type 2) is a bilateral, acquired macular disorder that typically manifests in middle-aged individuals. Circular, yellowish subretinal deposits distinguish a vitelliform lesion (VL) and are very rarely observed in conjunction with MacTel type 2. The presence of VL in cases of MacTel type 2 may further contribute to visual deterioration and lead to misdiagnosis. We report the case of a 54-year-old, otherwise healthy man with MacTel type 2 and unilateral VL, in whom the VL lesion progressed to macular atrophy during a three-year follow-up period.

## Introduction

Macular telangiectasia type 2 (MacTel type 2), also known as parafoveal telangiectasia, is an uncommon, bilateral, degenerative retinal disorder that predominantly affects the juxtafoveal area [1]. The condition is characterized by Müller cell loss, photoreceptor degeneration, and eventual disruption of the outer nuclear and ellipsoid layers, typically presenting between the fifth and seventh decades of life. Fundoscopic findings in MacTel type 2 can be subtle, particularly in the early stages of the disease. The initial clinical sign is often a grayish discoloration of the temporal perifoveal retina, accompanied by the loss of the foveal pit. Characteristic vascular alterations, such as ectatic capillaries and right-angled venules diving into the deeper retinal layers, may develop later [2-5].

Although the classical presentation of MacTel type 2 involves a lamellar macular hole-like appearance with cyst-like cavitations and vascular irregularities, a minority of patients exhibit atypical features such as vitelliform lesions (VL), which are round, yellowish, subretinal deposits resembling those observed in Best Disease or adult-onset foveomacular vitelliform dystrophy [6,7]. The pathogenesis remains unclear; however, it may be associated with the accumulation of degenerative material between the photoreceptor layers and the retinal pigment epithelium (RPE), secondary to Müller cell dysfunction and impaired shedding of retinal cells [2,8].

Vitelliform lesions in MacTel type 2 may potentially confound the clinical diagnosis by imitating other macular dystrophies or exudative processes, such as Best Disease or adult-onset foveomacular vitelliform dystrophy [9]. Hereby, we present an unusual case of MacTel type 2, in which the patient presented with a unilateral visual disturbance related to VL. The VL progressed to geographic atrophy over the longitudinal follow-up period, as evidenced by multimodal imaging.

## Case report

**Fig. 1:**
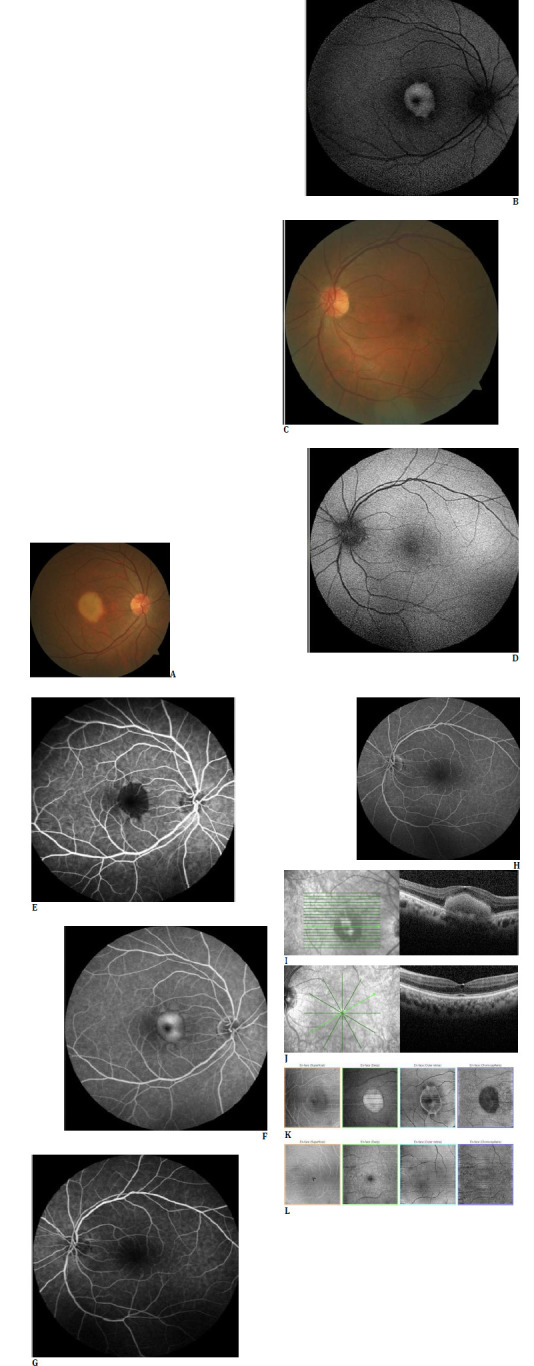
At presentation (March 2022): The color fundus photograph depicts a yellowish lesion of 1.5 disc diameters with sharply defined borders at the right posterior pole (**A**) and minimal foveolar color change at the left posterior pole (**C**). Autofluorescent image of the right eye revealing a hyperautofluorescent area with central hypoautofluorescence with well-demarcated borders (**B**). Almost normal-looking left autofluorescent image (**D**). Early (**E**) and late (**F**) right fluorescein angiograms demonstrating the staining of an early hypofluorescent-looking central lesion. Early (**G**) and late (**H**) left angiograms depicting the subtle late foveolar hyperfluorescence. Right spectral domain optical coherence tomography (SD OCT) showing the homogeneous hyperreflective subretinal lesion (**I**). Left SD OCT disclosing the subtle inner retinal cavity with an internal limiting membrane drape (**J**). Right en face optical coherence tomography (OCT) revealing a circular, well-demarcated lesion (**K**). Left enface OCT delineating a small hyporeflective focus, particularly noted on both superficial and deep slabs (**L**)

A 54-year-old, otherwise healthy man was referred to our clinic in March 2022 due to right visual deterioration. Upon examination, his best-corrected visual acuity was 20/200 in the right eye and 20/25 in the left eye. The slit-lamp examination was unremarkable except for mild bilateral nuclear sclerosis. Intraocular pressure was within normal limits in both eyes. Fundus examination of the right eye (Visucam® 500, Carl Zeiss Meditec AG, Germany) identified a yellowish foveal lesion of 1.5 disk diameters in size with well-defined borders (**Fig. 1A**). Additionally, a very subtle, small left foveolar color change was noted (**Fig. 1C**). The fundus autofluorescent image (Heidelberg Retinal Angiography 2) of the right eye revealed a centrally hypoautofluorescent area surrounded by a hyperautofluorescent ring with clearly defined margins (**Fig. 1B**). In contrast, the left foveal autofluorescence appearance was almost normal (**Fig. 1D**). The fluorescein angiogram of the right eye (Heidelberg Retinal Angiography 2) demonstrated an early hyperfluorescent central lesion with late staining, except for a small central hypofluorescent area (**Fig. 1E, F**). In contrast, only subtle late foveolar hyperfluorescence was observed in the left angiogram (**Fig. 1G, H**).

Spectral domain optical coherence tomography (OCT) (Spectralis OCT^®^, Heidelberg Engineering, Heidelberg, Germany) delineated elevated hyperreflective subretinal material in the right eye (**Fig. 1I**). In contrast, there was a slight foveolar inner retinal cavitation with internal limiting membrane drape in the left eye (**Fig. 1J**). On en face optical coherence tomography of the right eye (Triton, Topcon Inc., Oakland, New Jersey, USA), the circular deposition appeared hyperreflective on the deep layer slab and hyporeflective on the choriocapillaris slab (**Fig. 1K**). The left en face OCT showed a minute hyporeflective area in both the superficial and deep slabs (**Fig. 1L**). Our diagnosis was MacTel type 2 telangiectasia accompanied by a right macular vitelliform lesion, and we elected to monitor the patient without any treatment.

**Fig. 2:**
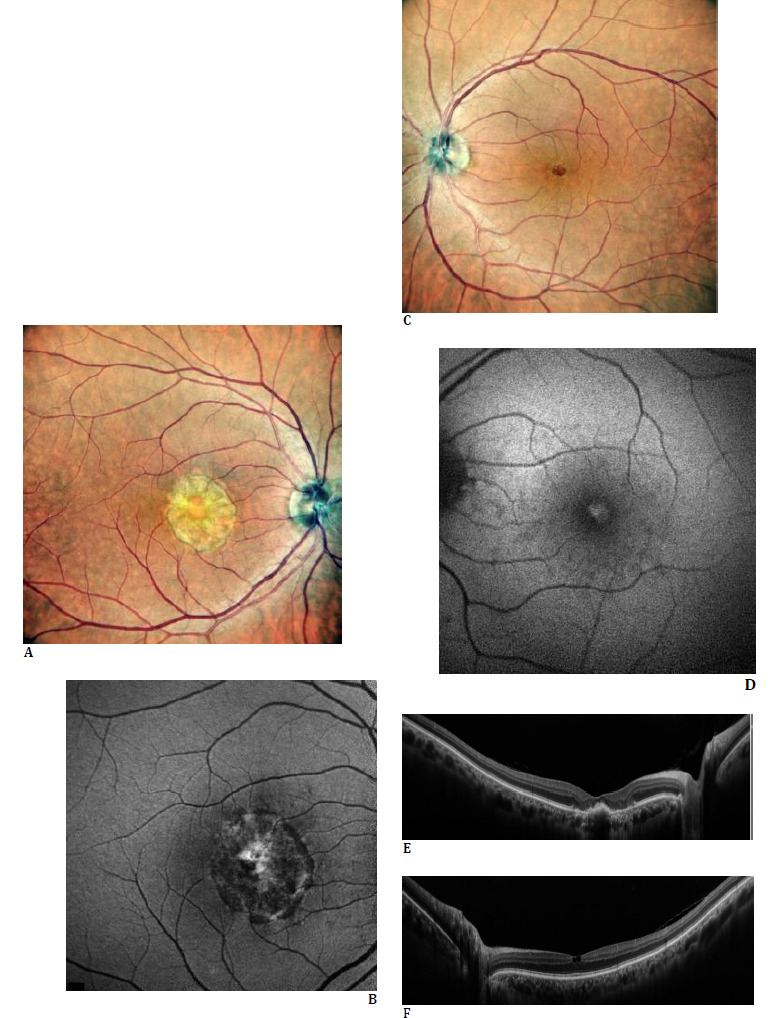
At follow-up visit (November 2023): Right color fundus picture revealing the well-demarcated yellowish homogenous lesion (**A**). Left color fundus image disclosing a minute central reddish discoloration (**C**). Right autofluorescent image demonstrating a small central hyperautofluorescence surrounded by a circular hypoautofluorescence (**B**), and a hyperautofluorescent minute area at the left fovea (**D**). Right swept-source optical coherence tomography (SS-OCT) scan delineating a decrease in height of the hyperreflective material (**E**) and the left SS-OCT section exhibiting the inner retinal cavity and internal limiting membrane drape (**F**)

In November 2023, the patient underwent re-examination, and no change in visual acuity was observed in either eye. Although the right eye exhibited a transformation of the homogeneous yellowish vitelliform material into a slightly patchy appearance, the left macula remained unaltered (**Fig. 2A, C**) (NIDEK Mirante; Nidek Co., Ltd., Gamagori, Japan). The right autofluorescent image (**Fig. 2B**) exhibited a small hyperautofluorescent area surrounded by a circular hypoautofluorescent zone, whereas the left autofluorescent image (**Fig. 2D**) revealed a foveolar hyperautofluorescence. Swept-source OCT sections demonstrated a reduction in the height of vitelliform material in the right eye, whereas the anatomy of the left fovea remained almost the same as in the initial OCT findings (**Fig. 2E, F**) (NIDEK Mirante; Nidek Co., Ltd., Gamagori, Japan).

**Fig. 3:**
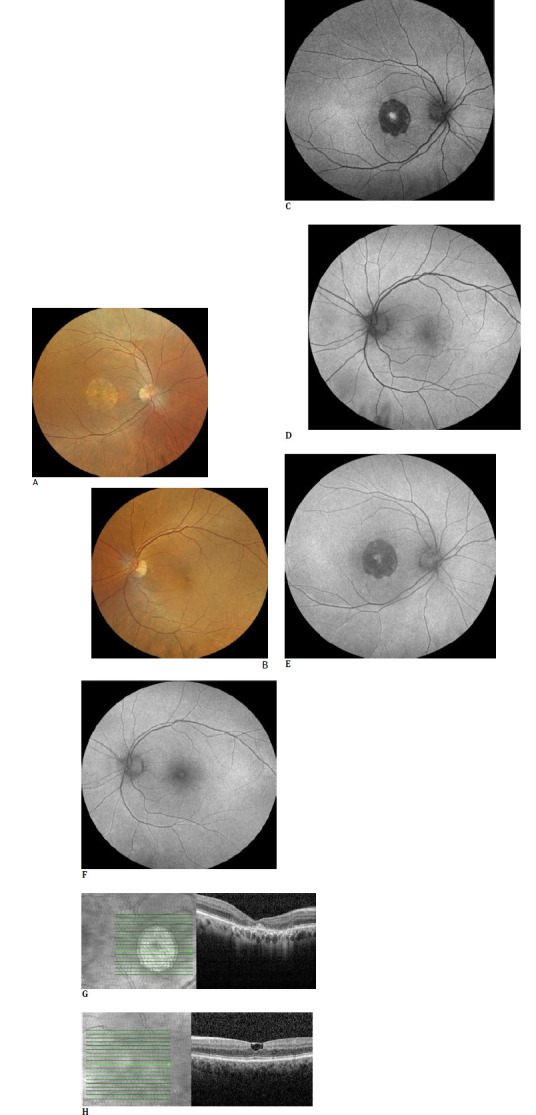
At last follow-up visit (June 2025): Color fundus pictures of the right eye (**A**) and left eye (**B**) depicting similar appearances compared to the previous visit. Green- and blue-autofluorescent images of the right eye (**C, E**) and left eye (**D, F**) again appear unchanged. Right SD-OCT section revealing a significant decrease in subretinal hyperreflective material height, along with the signs of atrophy (**G**). The OCT scan of the left eye looks unaltered (**H**)

In June 2025, the patient underwent another examination, including color fundus photography and blue- and green-autofluorescence imaging (Optos California RGB; Nikon, Dunfermline, Scotland). There was almost no change in the fundus appearance compared with the 2023 examination (**Fig. 3A-F**). Notably, on OCT, the height of the vitelliform deposition was markedly reduced, coinciding with the signs of foveal atrophy in the right macula, whereas no additional changes were observed in the left macula (**Fig. 3G, H**).

**Fig. 4:**
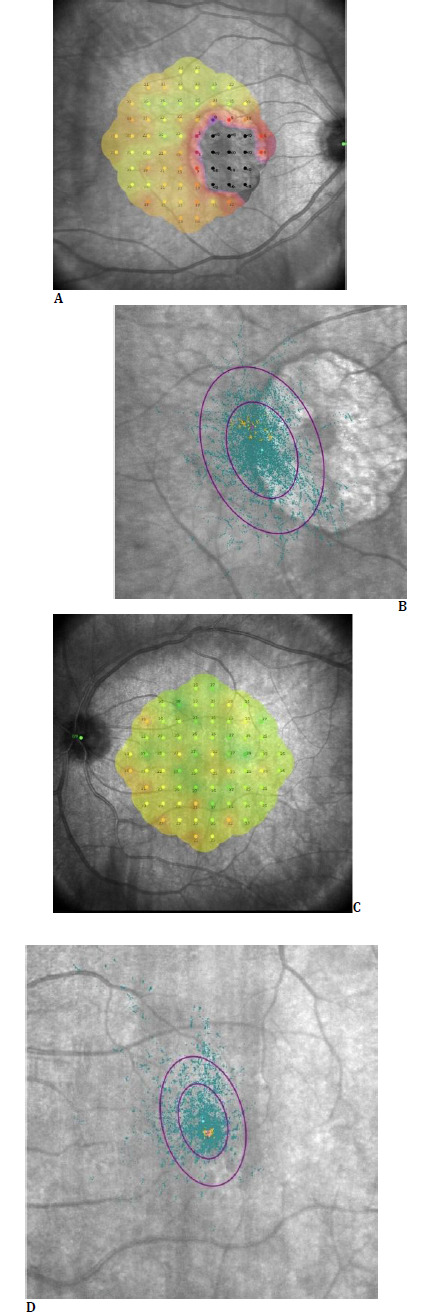
Microperimetry at the last visit (June 2025): Microperimetry images of the right (**A, B**) and left (**C, D**) eyes are shown. In the microperimetry image of the right eye (**A**), an absolute scotoma (black) is present at the location of the lesion. The retinal sensitivity map is color-coded (red: low sensitivity; green: high sensitivity); (**B**) The fixation map of the same eye shows that the turquoise dots represent fixation points, and the purple ellipses represent the BCEA 63 (small circle) and BCEA 95 (large circle) areas, respectively; (**C**) The microperimetry image of the left eye shows that retinal sensitivity is preserved; (**D**) The fixation points in the left eye are clustered in the central area, and fixation stability is higher than in the right eye. The purple ellipses also represent the BCEA 63 and BCEA 95 parameters

Microperimetric evaluation (MAIA, Macular Integrity Assessment, Centervue, Padova, Italy) revealed P1 and P2 values of 25% and 56%, respectively, in the right eye. The average threshold (AT) value was 15.6 dB. The eye with unstable fixation showed wide bivariate contour ellipse area (BCEA) values (BCEA 63 = 19.8°^2^, BCEA 95 = 54.9°^2^). An absolute scotoma was visible in the lesion area, with retinal sensitivity decreasing at the lesion edge and gradually increasing with distance from the lesion. In the left eye, the P1 value was 59%, the P2 value was 84%, and the AT value was 24.5 dB. The eye with relatively stable fixation had a BCEA 63 of 7.4°^2^ and a BCEA 95 of 22.3°^2^ (**Fig. 4 A-D**).

## Discussion

Vitelliform lesions are infrequently observed in MacTel type 2 [6,8,9]. Margalit et al. [7] described a unilateral, small lesion that the authors termed pseudovitelliform material in a 48-year-old woman presenting with MacTel type 2 and basal laminar drusen. The lesion was blocking the fluorescence. Nevertheless, no subsequent follow-up was documented, and the report dates back to the pre-OCT era. Lekha et al. [9] documented a 71-year-old woman presenting with a yellowish, submacular, vitelliform-like lesion approximately one-third of the disc diameter in the right eye and a typical MacTel type 2 appearance in the left eye. OCT of the right eye revealed subfoveal hyperreflective clumps of echoes and discontinuity of the ellipsoid line with intact retinal pigment epithelium (RPE). The vitelliform lesion remained unchanged both clinically and on OCT images. It was discussed that a reactive RPE response, manifested as hypertrophy, hyperpigmentation, or anterior migration, was observed in cases of adult-onset foveomacular vitelliform dystrophy. In contrast, MacTel type 2 with VL did not exhibit such alterations. Furthermore, the material exhibited hypofluorescence during the initial stages of fluorescein angiography and demonstrated late staining in cases of adult-onset foveomacular vitelliform dystrophy; however, these characteristics were not observed in MacTel type 2 with VL [9]. Pradhan et al. [10] recently reported two cases of MacTel type 2 associated with vitelliform lesions. The first case involved a 35-year-old female presenting with right-sided MacTel type 2 and a vitelliform lesion, with a normal left fovea. The second case was a 48-year-old male with MacTel type 2 and bilateral acquired vitelliform lesions. However, no follow-up was conducted in either case.

In previously documented cases involving MacTel type 2 and vitelliform lesions, no significant follow-up was reported. The current case had a three-year follow-up with comprehensive multimodal imaging analysis, demonstrating progression of the unilateral vitelliform lesion to foveal atrophy. Although the prognostic significance of vitelliform lesions in MacTel 2 remains unclear, their presence may be fraught with an increased risk of imminent macular atrophy and other complications, regardless of the accompanying pathology [11].

## Conclusions

The vitelliform lesion is an uncommon feature of MacTel type 2, and its presence may indicate a poor visual prognosis. Accurate clinical assessment can be achieved only through multimodal imaging to distinguish overlapping entities. We assert that this case underscores the importance of high-resolution imaging techniques for diagnosing and monitoring vitelliform lesions in patients with MacTel type 2.
